# From Thomas Willis to New Delhi

**DOI:** 10.4103/0972-2327.42931

**Published:** 2008

**Authors:** Sanjeev V. Thomas

**Affiliations:** Editor, Annals of Indian Academy of Neurology, Department of Neurology, Sree Chitra Tirunal Institute for Medical Sciences and Technology, Trivandrum, India

Thomas Willis described Myasthenia Gravis in a woman who had episodic muscular weakness, for the first time in 1672. In 1934, another English doctor, Mary Walker demonstrated that the weakness due to Myasthenia could be reversed by injection of Physostigmine, which was then popular as an antidote to curare poisoning. Sir Henry Dale, who was awarded the Nobel Prize for Medicine and Physiology in 1936, developed the neurohumoral theory and postulated on the acetyl choline receptors. Much progress has taken place since then. Now we know that neuromuscular junction disorders could be due to a variety of pathological processes. The neurophysiological and immunological basis of these disorders has been elaborated much. Prof. Angela Vincent has provided an excellent update on this subject in her Prof. Baldev Singh Oration. We are glad to bring out this oration which she had delivered in the 15^th^ Annual Meeting of the Indian Academy of Neurology in Mumbai, last year.

We have published several important articles on stroke in this journal recently. Stroke is one of the most important causes of mortality and disability in the world. Carotid stenosis is an important etiological factor for ischemic strokes in adults. Dr. Michael Ellis DeBakey made history in 1953 by performing the first successful carotid endarterectomy in the Methodist Hospital, Houston Texas, USA. Now more than 150,000 such procedures are being carried out in USA alone. Over the years several major studies have confirmed that carotid endarterectomy, when performed on critically stenosed artery protects from stroke. Recently interventional intravascular procedures have come up as possible alternative to endarterectomy. There are 29 clinical trials registered with the NIH Clinical Trial registry that examine various therapeutic aspects of carotid artery disease. Some studies are focusing on asymptomatic stenosis, while others are focusing on secondary prophylaxis. Certain studies are comparing endarterectomy with stent procedures. In this issue, we have included the long term experience of carotid endarterectomy of a single Institution in India.

This issue of the Annals will be reaching you a few weeks before the Annual Meeting of the Indian Academy of Neurology. This year, the annual meeting will be held in New Delhi, jointly with the 12^th^ Asia Oceanian Congress of Neurology. Earlier, we have had several excellent meetings in New Delhi. I am confident that the organizers of this meeting will exceed the performance of all previous meetings and provide us with a very informative and enjoyable meeting. I look forward to meet some of you on this occasion.

